# Interaction of Normal and Sickle Hemoglobins for Sodium Dodecylsulphate and Hydrogen Peroxide at pH 5.0 and 7.2

**DOI:** 10.1155/2013/629640

**Published:** 2013-10-10

**Authors:** Fortunatus C. Ezebuo, Sabinus Oscar O. Eze, Colin B. Lukong, Ferdinand C. Chilaka

**Affiliations:** ^1^Department of Biochemistry, University of Nigeria, Nsukka, Enugu State, Nigeria; ^2^Department of Biochemistry, Anambra State University, Uli, Nigeria

## Abstract

Clinical manifestations of malaria primarily result from proliferation of the parasite within the hosts' erythrocytes. The malaria parasite digests hemoglobin within its digestive vacuole through a sequential metabolic process involving multiple proteases. The activities of these proteases could lead to the production of ROS which could lead to the death of the parasites due to the destruction of their membrane. The action of SDS on hemoglobins can be likened to the way malarial proteases destabilizes host hemoglobin. Hence, the study was designed to determine the binding parameters of SDS and H_2_O_2_ for normal, sickle trait carrier and sickle hemoglobins at pH 5.0 and 7.2 using UV-VIS Titration Spectrophotometry. Hb-SDS interactions were significantly different at pH 5.0 but were not at pH 7.2. Also, Hb-H_2_O_2_ interactions were statistically different at pH 5.0 and 7.2. The interactions suggest that HbA and HbS are easily destabilized than HbAS and that HbAS has more affinity for H_2_O_2_. These suggest a production of more ferryl intermediates or hydroxyl radicals. All these interactions may hinder the development of the malaria parasite at the intraerythrocytic stage and could likely account for a significant proportion of the mechanism that favours the resistance to malaria by individuals with HbAS.

## 1. Introduction

Malaria is one of the most important infectious disease problems of humans, particularly in developing countries. *Plasmodium falciparum*, the most virulent human malaria parasite, is responsible for hundreds of millions of illnesses and more than one million deaths each year [1]. Clinical manifestations of malaria primarily result from proliferation of the parasite within the hosts' erythrocytes. During this process, hemoglobin is utilized as the predominant source of nutrition. This is because during the intraerythrocytic development and proliferation, the parasites ingest more than 75% of the hosts' hemoglobin and digest them within the digestive vacuole—an acidic organelles with estimated pH of 5.0–5.4—through a sequential metabolic process involving multiple proteases [2, 3], and action of sodium dodecylsulphate (SDS) on hemoglobins can be likened to the way proteases secreted by malaria parasites destabilizes host hemoglobin for their homeostasis. Hydrogen peroxide is a major reactive oxygen species in living organisms and can produce reactive hydroxyl radicals or ferryl intermediate [Fe(IV)=O]^2+^ by Fenton or Fenton like reaction [4]. Hanspal et al. [5] reported that *Plasmodium falciparum*-derived cysteine protease, falcipain-2, cleaves host erythrocyte hemoglobin at acidic pH and specific components of the membrane skeleton at neutral pH. Invasion of erythrocytes by plasmodium merozoites is a complex multistep process which is mediated by specific molecular interactions between host receptors and parasite ligands. A clear understanding of the molecular mechanisms involved in erythrocyte invasion and proliferation of the parasite could lead to the development of novel approaches to inhibit invasion, limit blood-stage parasite growth, and protect against malaria [6]. 

Molecular recognition lies at the heart of biological processes, and much effort is being made by biological chemist to understand the molecular details of macromolecule-ligand interactions of which hemoglobin-SDS and hemoglobin-H_2_O_2_ are not exception. Also, medical chemists are trying to exploit this understanding in developing useful pharmaceutics [7]. Dissociation constant (*K*_*d*_) is a reciprocal of binding constant (*K*_*b*_), and it is a useful way to present the affinity of a ligand for a macromolecule. This is because its value quickly tells us the concentration of ligand that is required to yield a significant amount of interaction with the macromolecule. The precise mechanism by which sickle cell trait imparts resistance to malaria is unknown. Hence, investigation was carried out on affinities of hemoglobins for SDS and H_2_O_2_, which could provide insight to mechanism that favours the resistance to malaria by individuals with HbAS variant.

## 2. Materials and Methods

### 2.1. Materials

Sodium dodecylsulphate (SDS) and other chemicals used in this work obtained from BDH, England and Sigma, Germany are of analytical grade. All reagents were freshly prepared unless otherwise stated.

### 2.2. Methods

Four milliliters (4 mL) of blood samples were collected from each of the identified individuals of genotype AA, AS, and SS after informed consent. In each case, the blood sample was collected with an ethylene di-amine tetra acetic acid (EDTA) vial. 

#### 2.2.1. Isolation and Purification of Hemoglobin

Each of the blood samples was combined with normal saline in 50 mM Tris-HCl (pH 7.2) in the ratio of 2 : 3 and centrifuged at 4°C for 10 min at 4000 rpm [8]. Thereafter, the supernatants were removed by aspiration. The centrifugation was repeated for 2–4 times until a clear supernatant is gotten in each case. The clear supernatants were removed and the resulting pellets in the case were made up to 5 mL with 50 mM Tris-HCl (pH 7.2). The red cells were lysed and 5% NaCl was added to the resulting volume and centrifuged for 10 min at 4000 rpm to remove inorganic phosphates and other ions. The crude hemoglobins were collected with separate vials and labeled appropriately. Each of the crude hemoglobins (HbA, HbAS, or HbS) was dialyzed at 4°C for 24 hr against 50 mM Tris-HCl buffer, pH 7.2. The dialyzed hemoglobins were collected and stored at –20°C for further experiments. 

#### 2.2.2. UV-Visible Titration

One hundred microliters (100 *μ*L) of 0.01 mM of each of the hemoglobins calculated on heme basis by using *ε*_415_ = 1.25 × 10^5^ M^−1^ cm^−1^ [9], or *ε*_523_ = 7.12 mM^−1^ cm^−1^ [10] were scanned from 250–650 nm using JENWAY 6405 UV-VIS Spectrophotometer in the absence and presence of different concentrations of ligands (sodium dodecylsulphate and hydrogen peroxide) in 50 mM buffers of pH 5.0 and 7.2 after appropriate buffer baselines. The titrations were done by fixing 0.1 mL of the hemoglobins in 3 mL cuvette containing a fixed volume of the buffer (2.1 mL each for SDS and H_2_O_2_) then various volumes (0 to 0.4 mL) corresponding to different in situ concentrations of SDS and H_2_O_2_ (0 to 0.748 mM) were added in stepwise manner from stock concentration of SDS or H_2_O_2_ (5.0 mM) mixed and scanned from 250–650 nm. Spectrum readings were recorded at each titration point (after each addition of SDS or H_2_O_2_). 

#### 2.2.3. Calculation of Dissociation Constant

The binding of Sodium dodecylsulphate (SDS) or Hydrogen peroxide (H_2_O_2_) to hemoglobin can be represented as follows:

(1) $$\begin{array}{c} \text{Hb}+n\text{L}⇌\text{HbL}n, \end{array}$$

where Hb represents HbA, HbAS, or HbS; L represents SDS or H_2_O_2_; *n* also known as *h* represents number of binding sites of SDS or H_2_O_2_ for hemoglobin.

*K*_*d*_, the dissociation constant is defined as

(2) $$\begin{array}{c} K_{d}=\frac{\;\left[\text{Hb}\right]{\left[\text{L}\right]}^{n}}{\left[\text{HbL}n\right]}, \end{array}$$

where [Hb] and [L] are the concentrations of free hemoglobin and ligands (SDS or H_2_O_2_) and [HbL*n*] is the concentration of the complex. The ratio of the concentration of the complexed ligand (SDS or H_2_O_2_), [HbL*n*], versus the total concentration of the hemoglobin,  [Hb]_o_  is denoted as *α*:

(3) $$\begin{array}{c} \alpha =\frac{\left[\text{HbL}n\right]}{{\left[\text{Hb}\right]}_{\text{o}}}=\frac{\left[\text{HbL}n\right]}{\left[\text{Hb}\right]+\left[\text{HbL}n\right]}. \end{array}$$

In this experiment,  [Hb]_o_  is fixed. From (2),  [HbL*n*] = [Hb][L]^*n*^/*K*_*d*_, hence,

(4) $$\begin{array}{c} \alpha =\frac{\left(\left[\text{Hb}\right]{\left[\text{L}\right]}^{n}/K_{d}\right)}{\left[\text{Hb}\right]+\left(\left[\text{Hb}\right]{\left[\text{L}\right]}^{n}/K_{d}\right)}. \end{array}$$

Dividing both numerator and denominator of (4) by  [Hb]  and multiplying by *K*_*d*_ gives

(5) $$\begin{array}{c} \alpha =\frac{{\left[\text{L}\right]}^{n}}{K_{d}+{\left[\text{L}\right]}^{n}}. \end{array}$$

From (5), a plot of *α* versus [L] gives a hyperbolic graph. When  *α* = 0.5, [L] = *K*_*d*_. To determine the *K*_*d*_ for Hb-SDS and Hb-H_2_O_2_ interactions, the increase in absorbance intensity at 415 nm upon addition of SDS (pH 5.0) or the decrease in absorbance intensity at 415 nm upon addition of SDS (pH 7.2) or H_2_O_2_ (pH 5.0 and 7.2), absorbance difference (Δ*A*) was used as an indication of the formation of [HbL*n*] complex. The value of Δ*A* for Hb-SDS interaction at pH 5.0 was calculated as *A* − *A*_o_ while that for Hb-SDS (pH 7.2) and Hb-H_2_O_2_ (pH 5.0 and 7.2) interactions were calculated as *A*_o_ − *A*. Where  *A*_o_  is absorbance at zero concentration of ligand and *A* is absorbance at *i*th concentration of the ligand.

The value of *α* was determined from

(6) $$\begin{array}{c} \frac{\Delta A}{{\;\Delta A}_{\max}}=\alpha , \end{array}$$

where Δ*A*_max⁡_ is the maximum change in the absorption intensity at 415 nm when SDS or H_2_O_2_ is saturated with Hb. It represents a fraction of a limiting compound of the system (in this case Hb) that binds to a compound with surplus concentration (in this case SDS or H_2_O_2_):

(7) $$\begin{array}{c} \frac{\Delta A}{{\Delta A}_{\max}}=\alpha =\frac{{\left[\text{L}\right]}^{n}}{K_{d}+{\left[\text{L}\right]}^{n}}. \end{array}$$

Because the experiment was performed using a concentration of ligand (SDS or H_2_O_2_) in the *K*_*d*_ range,  [L] ≈ [L]_o_, the total concentration of SDS or H_2_O_2_. This allows a simple approximation by substituting [L] with  [L]_o_, and (7) can be modified to

(8) $$\begin{array}{c} \Delta A=\frac{{\Delta A}_{\max}{\left[\text{L}\right]}_{\text{o}}^{n}}{K_{d}+{\left[\text{L}\right]}_{\text{o}}^{n}}. \end{array}$$

A plot of Δ*A* versus [L]_o_ was used to estimate Δ*A*_max⁡_. Then, Δ*A*_max⁡_ was used for calculating *α* values according to (6). The actual concentration of L in each titration can be calculated from

(9) $$\begin{array}{c} \left[\text{L}\right]={\left[\text{L}\right]}_{\text{o}}-\left[\text{HbL}n\right]={\left[\text{L}\right]}_{\text{o}}-\alpha {\left[\text{Hb}\right]}_{\text{o}} \end{array}$$

from (3), [HbL*n*] = *α*[Hb]_o_. [L]_o_ is known, thus,  [HbL*n*]  and [L] can be calculated accordingly [11]. The direct plot of  *α*  versus [L] according to (7) was used for the determination of *K*_*d*_ using nonlinear least-square regression analysis (*P* ≤ 0.05), a statistical package in GraphPad Prism version 5.04. 

## 3. Results and Discussion

**Figure 1:**
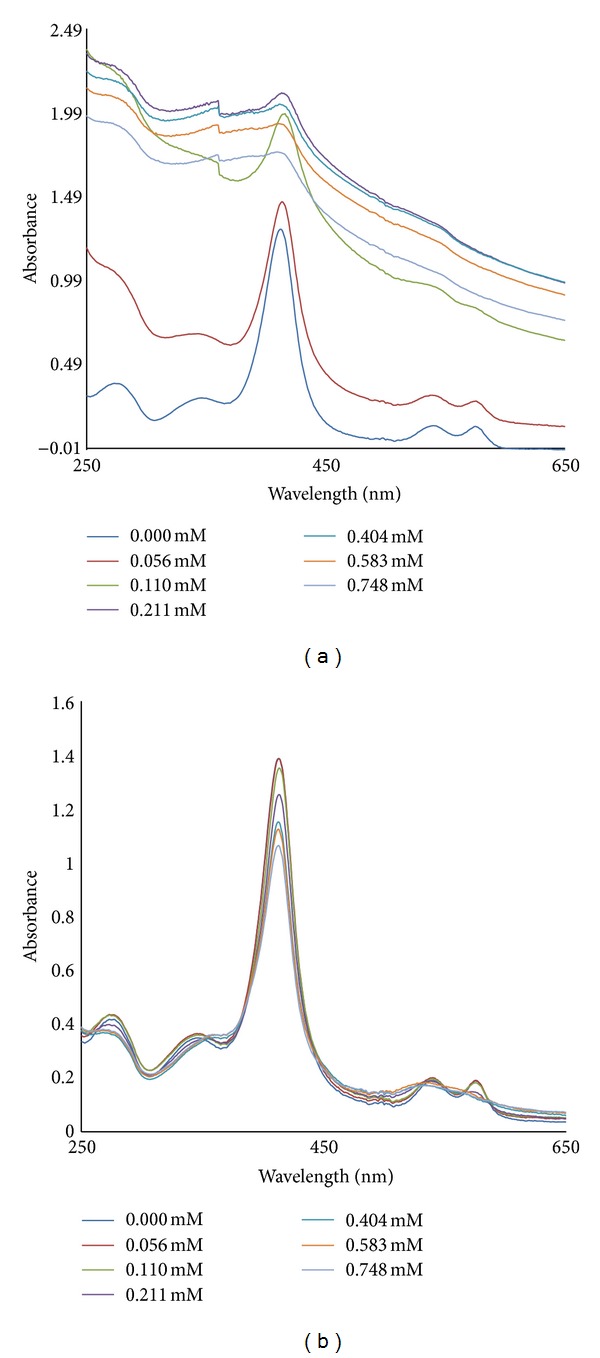
Absorption spectra of the titration: (a) pH 5.0 (b) pH 7.2: a solution of 0.01 mM Hb (0.1 mL) in 50 mM Tris-HCl buffer, 7.2 or Sodium acetate buffer, pH 5.0, was titrated with different concentrations (0–0.748 mM) of SDS, and spectrum readings were recorded at each titration point.

**Figure 2:**
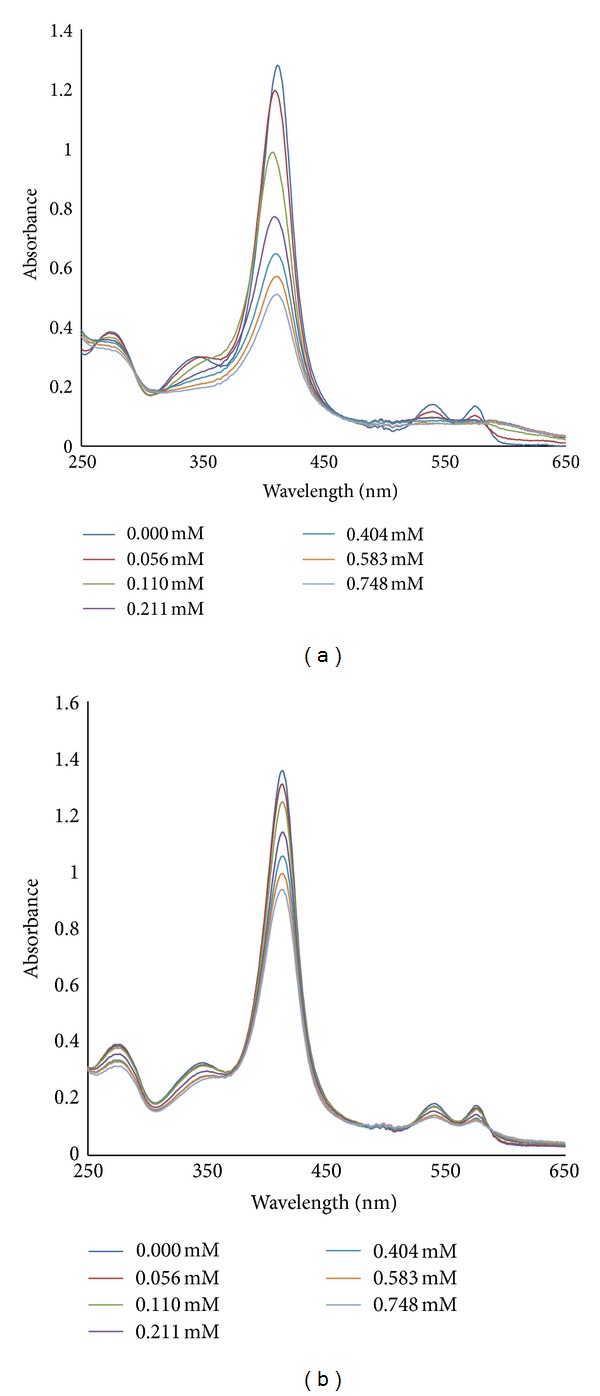
Absorption spectra of the titration: (a) pH 5.0 (b) pH 7.2: a solution of 0.01 mM Hb (0.1 mL) in 50 mM Tris-HCl buffer, 7.2 or Sodium acetate buffer, pH 5.0, was titrated with different concentrations (0–0.748 mM) of H_2_O_2,_ and spectrum readings were recorded at each titration point.

Titration of 0.1 mL of 0.01 mM of hemoglobins with SDS or H_2_O_2_ (0–0.748 mM) was monitored by absorption spectroscopy as shown in Figure 1. The titration was performed at pH 5.0 and 7.2. The absorption peak of hemoglobin at Soret [(hem-hem interaction), 400–420 nm] and aromatic (250–280 nm) bands increased upon adding increasing concentrations (0–0.748 mM) of SDS (pH 5.0) while the absorption peak decreased upon adding increasing concentrations (0–0.748 mM) of SDS or H_2_O_2_ (pH 5.0 and 7.2) (Figure 2). The increase in the absorbance of the aromatic band refers to dynamic motion of the molecule and its deviation from normal structure and function [12, 13], or unfolding of the hemoglobins [14, 15]. These can be likened to destabilization of hemoglobin structure by proteases such as plasmepsins and falcipains in the acidic environment of malaria parasite food vacuole as a result of malaria parasite infection. This unfolding exposes the heme moiety and buried aromatic amino acids of the proteins which explains the increase in absorbance observed at the soret and aromatic bands of the hemoglobins by SDS (pH 5.0). The decrease in absorbance on these bands by SDS at pH 7.2 suggests that the hemoglobins are folding while that by H_2_O_2_ (pH 5.0 and 7.2) suggests depletion of their heme content.

**Figure 3:**
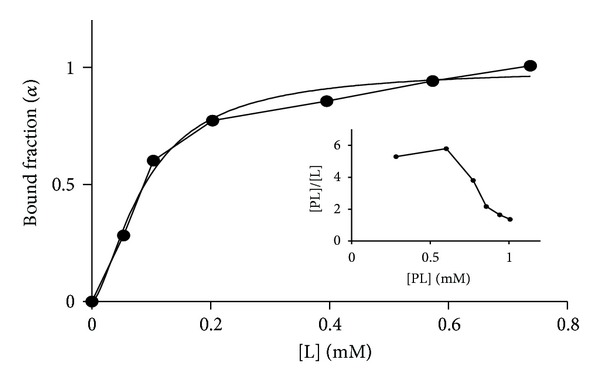
Nonlinear Least-square regression plot of *α* versus free L. Inset is a Scatchard plot of the experimental data.

The value of *K*_*d*_, Δ*A*_max⁡_, and *h* were determined from the plot of *α* versus the concentration of free ligand, L, ([SDS] or [H_2_O_2_]) as shown in Figure 3. The data were analyzed at *P* ≤ 0.05 according to (7) using nonlinear least-square regression, a statistical package in GraphPad. The calculated values of *K*_*d*_, Δ*A*_max⁡_, and *h* are shown in Table 1. At pH 5.0, the *K*_*d*_ of HbAS-SDS interaction was higher than that of HbA-SDS and HbS-SDS interactions. Action of SDS on hemoglobins can be likened to the way proteases secreted by malaria parasites destabilizes host hemoglobin for their homeostasis, therefore the result suggests that malaria parasite proteases easily destabilize HbA and HbS than HbAS at acidic pH. At pH 7.2, the *K*_*d*_ values calculated for HbA-SDS, HbAS-SDS and HbS-SDS were apparently the same (Table 1). The apparently same *K*_*d*_ values and decrease in absorption intensity at the soret band observed at increasing concentrations of SDS, pH 7.2 (Figure 1) suggests that malaria proteases dose does not destabilize (unfold) host hemoglobin (HbA, HbAS or HbS) at physiologic pH.

At pH 5.0 and 7.2, the calculated *K*_*d*_ values for HbAS-H_2_O_2_ interactions were smaller when compared with those calculated for HbA-H_2_O_2_ and HbS-H_2_O_2_ interactions (Table 1). Since smaller *K*_*d*_ means higher affinity, it implies that HbAS has more affinity for H_2_O_2_ than HbA and HbS at pH 5.0 and 7.2 with the affinity being more at pH 5.0. This suggests that HbAS-H_2_O_2_ interaction at pH 5.0 could produce more ferryl intermediate [Fe(IV)=O]^2+^ or hydroxyl radical (HO^∙^) by fenton or fenton-like reaction which may kill the malaria parasites at the intraerythrocytic stage of their development. This may not be the case with HbS due to hemolytic crisis which can even lead to invasion of more tissues by the malaria parasites.

Hb-SDS interactions (pH 5.0 and 7.2) and Hb-H_2_O_2_ interaction (pH 5.0) show positive cooperativity with Hb-SDS interactions at pH 5.0 being more cooperative while Hb-H_2_O_2_ interaction at pH 7.2 show no cooperativity (Table 1). This implies that binding of SDS or H_2_O_2_ to Hb at pH 5.0 or 7.2 brings about binding of more SDS or H_2_O_2_ to Hb while the binding of H_2_O_2_ to Hb at pH 7.2 does not cause binding of more H_2_O_2_ to Hb. It was also observed that Δ*A*_max⁡_ calculated for both Hb-SDS and Hb-H_2_O_2_ were approximately the same (Table 1).

## 4. Conclusion

Action of SDS on hemoglobins can be likened to the way proteases secreted by malaria parasites destabilizes host hemoglobin for their homeostasis. The higher *K*_*d*_ for HbAS-SDS interaction (pH 5.0) suggest that HbA and HbS are easily destabilize more than HbAS while the higher affinity of HbAS for H_2_O_2_ (pH 5.0) suggests the production of more ferryl intermediates or hydroxyl radicals. All these interactions may hinder the development of the malaria parasite at the intraerythrocytic stage and could likely account for a significant proportion of the mechanism that favours the resistance to malaria by individuals with the HbAS hemoglobin variant.

## Figures and Tables

**Table 1:** Interaction parameters of Hb with SDS and  H_2_O_2_ at pH's 5.0 and 7.2.

Hemoglobin sample				
Treatment	Parameters	HbA	HbAS	HbS
SDS, pH 5.0	Δ*A*_max⁡_	1.000 ± 0.005577	1.000 ± 0.006371	1.000 ± 0.05466
	*h* (*n*)	4.081 ± 2.382	4.954 ± 2.025	6.050 ± 4.771
	*K*_*d*_ (mM)	0.05641 ± 0.006	0.07368 ± 0.0097	0.05977 ± 0.0072
	*R* ^2^	0.9536	0.9479	0.9567

SDS, pH 7.2	Δ*A*_max⁡_	0.9963 ± 0.044	0.9951 ± 0.1448	0.9945 ± 0.09589
	*h* (*n*)	2.232 ± 0.2384	1.481 ± 0.2750	1.824 ± 0.3063
	*K*_*d*_ (mM)	0.2325 ± 0.016	0.2873 ± 0.07246	0.2547 ± 0.03908
	*R* ^2^	0.9974	0.9929	0.9938

H_2_O_2_, pH 5.0	*A*_max⁡_	0.9959 ± 0.06882	0.9968 ± 0.05220	0.9965 ± 0.08263
	*h* (*n*)	1.552 ± 0.2906	1.558 ± 0.3135	1.428 ± 0.3145
	*K*_*d*_ (mM)	0.1329 ± 0.01874	0.08883 ± 0.01005	0.1242 ± 0.02161
	*R* ^2^	0.9915	0.9909	0.9893

H_2_O_2_, pH 7.2	*A*_max⁡_	0.9954 ± 0.2002	0.9946 ± 0.08353	0.9963 ± 0.3365
	*h* (*n*)	1.123 ± 0.1706	1.570 ± 0.2138	1.121 ± 0.2203
	*K*_*d*_ (mM)	0.4310 ± 0.1681	0.2364 ± 0.03510	0.5772 ± 0.3536
	*R* ^2^	0.9963	0.9959	0.9941
